# COVID-19 in a patient with new adult-onset Still disease: A case report

**DOI:** 10.1097/MD.0000000000030953

**Published:** 2022-10-07

**Authors:** Samar O. Alharbi

**Affiliations:** a Department of Medicine, Taibah University, Medina, Saudi Arabia.

**Keywords:** adult-onset Still disease, case report, corticosteroids, COVID-19, hyperferritinemia

## Abstract

Adult-onset Still disease (AOSD) is a systemic autoinflammatory illness of unknown cause. Its manifestations comprise fever; arthritis or arthralgia; and skin rash with high inflammatory markers and ferritin levels. Coronavirus disease 2019 (COVID-19) shares several clinical features and laboratory markers of AOSD: making it challenging to differentiate between the 2 conditions.

**Patient concerns:**

A 29-year-old woman presented with fever, skin rash, and polyarthritis 4 weeks before admission. Two weeks after illness onset, she had an infection with symptoms similar to those of COVID-19. She observed that her symptoms worsened, and new symptoms appeared including headache; vomiting; diarrhea; and loss of taste and smell. The patient tested positive for severe acute respiratory syndrome coronavirus 2 using polymerase chain reaction.

**Diagnosis:**

The patient was diagnosed with AOSD complicated with COVID-19 after exclusion of other possible causes of her illness, such as infections, malignancy, or underlying rheumatological disease.

**Interventions:**

The patient was administered corticosteroids and methotrexate. The patient responded quickly, particularly to corticosteroids.

**Outcomes:**

This is the second reported case of COVID-19 in a patient with AOSD. She experienced COVID-19 shortly after having AOSD, indicating that those with AOSD might have a higher risk of COVID-19 infection. Furthermore, she developed the most prevalent COVID-19 symptoms. However, distinguishing most of these symptoms from AOSD manifestations was difficult.

**Lessons:**

Early diagnosis and differentiation between AOSD and COVID-19 and prompt initiation of treatment are required.

## 1. Introduction

“Still disease” was named after George Still who, in 1897, described 22 children with systemic onset juvenile idiopathic arthritis.^[1]^ In 1971, Eric Bywaters identified adult-onset Still disease (AOSD) by describing 14 adult patients with skin rash, fever, and polyarthritis whose clinical presentation closely resembled that of pediatric Still illness.^[2]^ AOSD is a rare autoinflammatory disorder of unknown etiology with an incidence of 0.16 to 0.40 per 100,000 individuals. However, its prevalence rate has been reported to be 1 to 34 cases per million people.^[3,4]^

The coronavirus disease 2019 (COVID-19) pandemic started in 2019 with symptoms ranging from asymptomatic to multi-organ failure. Numerous clinical and biochemical characteristics of AOSD and COVID-19 are similar. Both diseases are marked by high levels of serum ferritin and a process of hyperinflammation caused by a cytokine storm that can lead to multiple organ failure. The coexistence of both conditions, particularly when a patient with undiagnosed AOSD has COVID-19 infection, can make appropriate diagnosis very challenging. However, early diagnosis and treatment are crucial to avoid life-threatening complications.

Occurrence of COVID-19 in patients with AOSD remains to be elucidated. Therefore, we present the case of a recently diagnosed AOSD patient who acquired COVID-19 shortly after becoming ill. She had classic AOSD symptoms and was treated with corticosteroids and methotrexate (MTX). The patient responded rapidly, clinically, and biochemically, particularly to corticosteroids.

## 2. Case report

Our patient was a 29-year-old Saudi woman with a history of pseudotumor cerebri and acetazolamide use. The patient had no psychological or family history of autoimmune or rheumatological disease. She was admitted to the department of internal medicine in our hospital with chief complaints of high spiking fever reaching up to 39.5 °C combined with chills, skin rash, and polyarthritis that lasted for the last 4 weeks. Skin rash was non-itchy and transient erythematous maculopapular, mainly observed on her trunk and extremities. She also had joint pains involving the knee, ankle, shoulders, and wrists. Moreover, she showed recurrent sore throat and myalgia. Two days before admission, she experienced abdominal pain, vomiting, and diarrhea. During illness, she visited 2 medical centers and was treated with non-steroidal anti-inflammatory drugs, by which temporary mild relief was achieved. There was no clear diagnosis. At that time, the patient tested negative for severe acute respiratory syndrome coronavirus 2 (SARS-CoV-2) using nasopharyngeal swab reverse transcription polymerase chain reaction (RT-PCR) and further investigations were not requested. In addition, she received 2 COVID-19 vaccines 9 months before. Two weeks after her illness, she started to report headache, diarrhea, and loss of taste and smell. She was then suspected to have COVID-19. RT-PCR was repeated and revealed positivity for SARS-CoV-2. On the day of her admission, 10 days after testing positive for COVID-19, SARS-CoV-2 RT-PCR was repeated and showed negative results. The patient was then admitted to our department due to fever of unknown origin. She was unable to move due to myalgia and polyarthralgia. She continued to have a high-grade fever with subsided skin rash. She had no oral ulcers; hair loss; or cardiac, respiratory, or neurological symptoms. The patient also had mild abdominal pain but without vomiting or diarrhea.

On physical examination, she appeared ill and highly febrile (−39.4 °C). Oxygen saturation on room air was 98%. In addition, she had cervical lymphadenopathy. Skin examination was normal. Musculoskeletal examination showed tender joints without effusion. Abdominal, chest, cardiovascular, and neurological examinations revealed unremarkable findings. Laboratory test results revealed white blood cell count, 22.7 × 10^3^ cells/µL (4–10 × 10^3^ cells/µL); hemoglobin, 8 g/dL (12–15 g/dL); platelet, 671 × 10^9^/L (150–450 × 10^9^/L); erythrocyte sedimentation rate (ESR), 120 mm/hour (0–25 mm/hour); positive C-reactive protein; ferritin, 5616 ng/mL (13–150 ng/mL); lactate dehydrogenase, 490 U/L (100–247 U/L); and elevated liver enzymes aspartate transaminase, 69 U/L (<35 U/L) and alanine aminotransferase 72 U/L (<35 U/L). Renal function test, creatine kinase, triglyceride, fibrinogen, and peripheral blood smear were normal. Moreover, blood, urine, stool cultures, tuberculin skin test, Cytomegalovirus, Epistein–Barr virus, Human immunodeficiency virus, hepatitis B and C, malaria screen, *Salmonella* (Widal test), and *Brucella* serology tests were all negative. Rheumatology work-up including antinuclear antibodies, rheumatoid factor, anti-cyclic citrullinated peptide, anti-neutrophil cytoplasmic antibodies, and extractable nuclear antigen panel tests were also negative.

Thoracic echocardiogram showed no abnormalities. However, enhanced computed tomography scan of the neck showed mildly enlarged supraclavicular and cervical lymph nodes. Chest computed tomography scan showed bilateral small effusions and mild hepatosplenomegaly. Excisional lymph node biopsy was consistent with reactive lymphoid hyperplasia. Endoscopy and colonoscopy were unremarkable. The patient was examined by hematology, gastroenterology, and respiratory physicians and no abnormality was reported. Based on the above findings, she was finally diagnosed with AOSD.

The patient was initially administered intravenous prednisone (60 mg daily) and then switched to oral treatment with similar dose and improved dramatically. Her fever subsided and joint pain improved rapidly. Moreover, her laboratory abnormalities including complete blood count, liver enzymes, acute phase reactants, and ferritin improved. She was then discharged with oral prednisone 60 mg once a day to be tapered slowly. A plan was clearly explained to the patient. During her follow-up after 2 weeks, no fever, mild joint pain, and no skin rash or sore throat were found. Laboratory abnormalities showed further improvement. Her ESR and ferritin levels were 76 mm/hour and 1650 ng/mL, respectively. Oral MTX (15 mg) was administered once per week. A month after discharge, she improved without any signs or symptoms of the disease. The patient well tolerated MTX and started to taper off prednisone without any complications. Her ESR and ferritin level further improved to 40 mm/hour and 230 ng/mL, respectively. Despite improvement, she is still regularly and closely monitored during the follow-up period.

## 3. Discussion and conclusions

AOSD is a complex systemic autoinflammatory disorder that affects young male and female adults. Previous studies reported that female patients were more affected than male ones.^[5,6]^ The main clinical features of AOSD comprise fever; transient skin rash; and arthritis or arthralgia. Other features include sore throat, lymphadenopathy, serositis, splenomegaly, hepatomegaly, and increased levels of liver enzymes. Additionally, it is associated with hyperleukocytosis, high inflammatory markers, and high ferritin level.^[7,8]^ However, the pathophysiology of AOSD remains unclear. Multiple factors, including viral infections, genetics, and immunological dysregulation, have been linked to AOSD onset. Multiple factors have been linked to the start of AOSD, such as viral infections; genetics; and immunological dysregulation, such as inflammation caused by cytokines and dysregulated apoptosis.^[9–12]^

Due to the lack of specific laboratory tests that can distinguish AOSD from other conditions with similar symptoms, AOSD remains challenging to diagnose. Before appropriate diagnosis of AOSD, it is necessary to screen for infectious, neoplastic, and autoimmune illnesses. Our patient was admitted due to fever of unknown origin. Up to 20% of cases of fever of unknown cause can be attributable to AOSD. Moreover, approximately developing a high-spiking fever is one of the early clinical features of this disease.^[13,14]^ The Yamaguchi and Fautrel classification criteria (Table 1), often used for AOSD diagnosis, were met in our patient.^[15,16]^ This case was challenging because the patient had COVID-19 complicated with AOSD: symptoms of which had started recently but was not immediately diagnosed. Both conditions share common clinical and biochemical features. Serum ferritin is recognized as a particular diagnostic criterion for AOSD.^[7]^ Moreover, serum ferritin levels that are 5 times higher than the usual upper limit are shown to have a sensitivity of 80% and specificity of 46% for diagnosing AOSD.^[17]^

**Table 1:** AOSD diagnostic criteria.

Yamaguchi et al classification for AOSD	Fautrel et al classification for AOSD
Major criteria:	Major criteria:
1. Fever of 39 °C lasting at least 1 wk	1. Spiking fever 39 °C
2. Arthralgia or arthritis for 2 wk	2. Arthralgia
3. Typical nonpruritic salmon-pink skin rash	3. Transient erythema
4. Leukocytosis 10,000/mm^3^ with 80% polymorphonuclear cells	4. Pharyngitis
	5. Polymorphonuclear cells!80%
	6. Glycosylated ferritin 20%
Minor criteria:	Minor criteria:
1. Sore throat	1. Maculopapular rash
2. Lymph node enlargement	2. Leukocytes 10,000/mm^3^
3. Hepatomegaly or splenomegaly	
4. Abnormal liver function tests	
5. Negative ANA and RF tests	
Exclusion criteria:	
1. Infections (especially, sepsis and infectious mononucleosis)	
2. Malignancy (mainly malignant lymphoma)	
3. Other rheumatic disorders (mainly polyarteritis nodosa and rheumatoid vasculitis with extraarticular features)	
For diagnosis of AOSD, patient should meet “5 or more criteria, of which at least 2 should be major”	For diagnosis of AOSD, patient should meet “4 or more major criteria OR 3 major criteria + 2 minor criteria”.
Sensitivity: 96.2% Specificity: 92.1%	Sensitivity: 80.6% Specificity: 98.5%

AOSD = adult-onset Still disease.

AOSD, macrophage activation syndrome, catastrophic antiphospholipid syndrome, and septic shock are the 4 clinical conditions under the general term “hyperferritinemic syndromes”. They are all marked by elevated serum ferritin levels and cytokine storm that ultimately result in multi-organ failure.^[18]^ In addition, COVID-19 has been recently included in the definition of “hyperferritinemic syndromes” due to similar clinical characteristics and its associated complications.^[19]^ According to the findings of Colafrancesco et al, ferritin expression was higher in patients with AOSD than in those with COVID-19.^[20]^

Data on COVID-19 occurrence in patients with AOSD is obscure. As components of hyperferritinemic syndromes, COVID-19 and AOSD occur at different incidence rates. However, there is only 1 case reported in 2021 of a patient with AOSD who was in remission when he had COVID-19.^[21]^ To the best of our knowledge, this is the second case. However, our case is unique because our patient had AOSD symptoms 2 weeks before having COVID-19, which was not immediately diagnosed. On the other hand, several case reports showed an inappropriate immune response to COVID-19 that caused development of AOSD.^[22,23]^ Additionally, there are several reported cases of new-onset AOSD after COVID-19 vaccination.^[24–29]^

Severe COVID-19 and AOSD can be associated with life-threatening complications and high mortality rate when undiagnosed or not treated early. Similarities in the clinical presentation and laboratory markers make it challenging to diagnose and differentiate between the 2 conditions. Of note, the patient was admitted to our hospital and highly suspected of AOSD based on her initial symptoms, such as fever; skin rash; polyarthralgia; and negative SARS-CoV-2 PCR test, which was initially performed during her illness and repeated on admission. Moreover, detailed history was crucial because the patient was aware of the new symptoms of COVID-19 that started later and improved upon admission time with persistent and worsening of the other symptoms, particularly fever and arthralgia.

In conclusion, it has been reported that patients with autoimmune disorders may be more likely than the general population to be prone to COVID-19 infection. However, there is no available data regarding COVID-19 infection risk in patients with AOSD. Our patient developed COVID-19 shortly after having AOSD, indicating that those with AOSD might have a higher risk of acquiring COVID-19 infection. More research is required to establish the risk factors of COVID-19 infection in patients with AOSD. In addition, it is essential to determine if such diseases affect the clinical presentation of COVID-19 and the extent to which COVID-19 infection might affect the clinical course of AOSD. Our patient had the most prevalent COVID-19 symptoms, including sore throat, headache, myalgia, arthralgia, diarrhea, and loss of taste and smell. However, it was difficult to distinguish the majority of these symptoms from AOSD manifestations. Early diagnosis and treatment of such conditions is crucial to avoid poor outcomes.

## Acknowledgements

We would like to thank Editage (www.editage.com) for English language editing.

## Author contributions

**Conceptualization:** Samar Alharbi.

**Data curation:** Samar Alharbi.

**Formal analysis:** Samar Alharbi.

**Writing – original draft:** Samar Alharbi.

**Writing – review & editing:** Samar Alharbi.
